# Donor Commitments and Disbursements for Sexual and Reproductive Health Aid in Kenya, Tanzania, Uganda and Zambia

**DOI:** 10.3389/fpubh.2021.645499

**Published:** 2021-04-20

**Authors:** Denis Kibira, Cornelia Asiimwe, Moses Muwonge, Hendrika A. van den Ham, Tim Reed, Hubert G. Leufkens, Aukje K. Mantel-Teeuwisse

**Affiliations:** ^1^Division of Pharmacoepidemiology and Clinical Pharmacology, Faculty of Science, Utrecht Centre for Pharmaceutical Policy and Regulation, Utrecht Institute for Pharmaceutical Sciences, Utrecht University, Utrecht, Netherlands; ^2^Coalition for Health Promotion and Social Development (HEPS-Uganda), Kampala, Uganda; ^3^Samasha Medical Foundation, Kampala, Uganda; ^4^Health Action International, Amsterdam, Netherlands

**Keywords:** sexual and reproductive health and rights, development assistance for health, official development aid, donor aid, low-and-middle-income countries

## Abstract

**Background:** Sexual and Reproductive Health and Rights (SRHR) investments are critical to people's well-being. However, despite the demonstrated returns on investments, underfunding of SRHR still persists. The objective of this study was to characterize donor commitments and disbursements to SRH aid in four sub-Saharan countries of Kenya, Tanzania, Uganda and Zambia and to compare trends in donor aids with SRH outcome and impact indicators for each of these countries.

**Methods:** The study is a secondary analysis of data from the Organization for Economic Co-operation and Development's Assistance creditor reporting system and SRH indicator data from the Global Health Observatory and country demographic health surveys for a 16-year period (2002–2017). We downloaded and compared commitments to disbursements of all donors for population policies, programs and reproductive health for the four African countries. SRH indicators were stratified into health facility level process/outcome indicators (modern contraceptive prevalence rate, unmet need for family planning, antenatal care coverage and skilled birth attendance) and health impact level indicators (maternal mortality ratio, newborn mortality rate, infant mortality rate and under five mortality rate).

**Results:** Donor commitments for SRH aid grew on average by 20% while disbursements grew by 21% annually between 2002 and 2017. The overall disbursement rate was 93%. Development Assistance Cooperation (DAC) countries donated the largest proportion (79%) of aid. Kenya took 33% of total aid, followed by Tanzania 26%, Uganda 23% and then Zambia (18%). There was improvement in all SRH outcome and impact indicators, but not enough to meet targets.

**Conclusion:** Donor aid to SRH grew over time and in the same period indicators improved, but improvement remained slow. Unpredictability and insufficiency of aid may be disruptive to recipient country planning. Donors and low- and middle-income countries should increase funding in order to meet global SRHR targets.

## Background

Universal access to sexual and reproductive health and rights (SRHR) is necessary for the achievement of people's social, economic and environmental dimensions of sustainable development (1). The attainment of SRHR has not been realized as highlighted by an estimated annual death of more than 350,000 women and 5.6 million children worldwide from preventable complications related to pregnancy and childbirth (2).

Developing countries are affected disproportionately with 99% of the deaths from complications related to pregnancy and childbirth which could be mostly prevented by proper healthcare and services (3). Developing countries have the highest maternal, newborn and under-five mortality rates in the world (4). About 80 per cent of under-five deaths occur in two regions, that is sub-Saharan Africa and Southern Asia (2). Table 1 shows SRH indicators for some of the most affected sub-Saharan countries namely Kenya, Tanzania, Uganda and Zambia.

**Table 1:** Comparison of SRH indicators for Kenya, Tanzania, Uganda and Zambia (4–8).

**Context** **Variable**	**Kenya**	**Tanzania**	**Uganda**	**Zambia**	**Sub-Saharan Africa**	**Global**
2019 Population (in millions)	52.574	58.005	44.270	17.861	1,066,283	7,713, 468
Total Fertility Rate	3.9	5.2	5.4	4.7	4.9	2.5
Modern Contraceptive Prevalence Rate	53%	32%	38%	48%	26%	55%
Unmet need for Family Planning	18%	23%	28%	20%	24%	12%
Teenage Pregnancy Rate	15%	27%	25%	29%	–	44%
Maternal Mortality Ratio (per 100,000 live births)	362	556	336	252	546	216
Infant Mortality Rate (per 1,000 live births)	39	43	43	42	56	30.5
Under five mortality Rate (per 1,000 live births)	52	67	53	61	79	41

SRHR is one of the inequities that the Millennium Development Goals (MDGs) sought to address (9). Building on the MDGs, the Sustainable Development Goals (SDGs), agreed by 193 world leaders in 2015, are a 17-point plan to end poverty, combat climate change and fight injustice and inequality. SDG 3 aims to ensure healthy lives and promote well-being for all at all ages (10).

SDG 3 sets targets by 2030 which include: reduce the global maternal mortality ratio to <70 per 100,000 live births; end preventable deaths of newborns and children under 5 years of age, with all countries aiming to reduce neonatal mortality to as low as 12 per 1,000 live births and under-five mortality to as low as 25 per 1,000 live births; ensure universal access to sexual and reproductive health-care services, including family planning, information and education, and the integration of reproductive health into national strategies and programs.

To meet the above targets and improve health status, adequate health financing is essential (11). However, low- and middle-income countries (LMICs), in which resources are limited, also have inadequate health expenditure by governments (12). For example, in financial year 2009/10, the Kenyan government allocated about US$12.20 per person (equivalent to 5.4 % of the domestic budget) to health, and in Uganda the domestic budget was about US$11.20 per person equivalent to 7.4 % of the budget (13). This is against a backdrop of US$ 34 per person recommended by the WHO Commission on Macroeconomics and Health for governments to spend per year to provide a set of essential interventions (14). The limited spending on health by LMIC governments has meant that outside support is required (15). The magnitude of external funding on health as a percentage of total health expenditure has been significant, varying from 11 to 60% in over 28 sub-Saharan countries (16).

The United Nations (UN) Secretary-General's *Global Strategy for Women's, Children's and Adolescents' Health, 2016–2030* aims to catalyze the SDGs by mobilizing stakeholders including governments, donors/development partners, civil society, academia, healthcare providers and communities to scale up and prioritize high-impact interventions for strengthening health systems, integrating efforts across diseases and sectors as well as promoting human rights, gender equality and poverty reduction (9). In low-income countries, where much development assistance for health (DAH) is targeted, it made up 34.6% of total health spending in 2016 (17). DAH was estimated to total $37.6 billion in 2016, up 0.1% from 2015. However, after a decade of rapid growth from 2000 to 2010 (11.4% increase annually), DAH grew at only 1.8% annually between 2010 and 2016. SRHR is one of the priority areas financed by DAH from wealthier nations and international agencies (18).

In order to improve accountability for DAH, there has been increased efforts in resource tracking (19, 20). Studies have tracked trends and magnitude of donor funding to different areas of SRH that is reproductive, maternal, newborn, and child health (21), and sought to verify whether donor resources are better targeted to countries with the highest need (21). However, there is need to further explore what determines donor aid to recipient countries, priorities funded by donors within recipient countries, donor aid predictability (including whether donors disburse what they commit), how the donor aid is used by recipient countries, its effectiveness, and how donor aid influences funding of priorities by recipient countries (22).

This study sought to characterize donor predictability by examining their commitments and disbursements for SRH aid in four of the most affected countries in sub-Saharan Africa. The study therefore described the types of donors, the value and trends of their commitment and disbursement for SRH aid and matched the aid to changes in SRH indicators across the four countries in order to add to the body of knowledge on DAH accountability.

## Methods

### Data Sources and Definitions

The study is a secondary analysis of data on donor aid commitments and disbursements for SRH from the Organization for Economic Co-operation and Development's Assistance creditor reporting system (OECD CRS) for a 16-year period (2002–2017).

The OECD CRS is a database to which donors of official development assistance (ODA), other official flows and private grants report their commitment and disbursement activities as described at http://www.oecd.org/dac/stats/methodology.html. The CRS is a publicly accessible web-based database on aid activities, developed and maintained by the Development Assistance Committee (DAC) of the OECD (18). OECD DAC commitments and disbursements are tracked at both the aggregate level and at the level of particular aid programmes (22).

ODA refers to grants or loans from members of the OECD DAC (a group of 30 nations including most of the West European and North American countries, the European Union, Australia, New Zeeland, Japan, and Korea), non-DAC bilateral donors (mostly Eastern European and Middle Eastern countries for example Croatia, Bulgaria, Turkey, Israel, United Arab Emirates, Kuwait), multilateral institutions (for example International Monetary Fund, regional development banks), global health initiatives (for example Global Fund to Fight Tuberculosis, AIDS and Malaria, Global Alliance for Vaccines and Immunization) and private philanthropists (for example Bill and Melinda Gates Foundation, Metlife Foundation, United Postcode Lotteries) with promotion of economic development and welfare as the main objective (22). In addition to financial flows, technical co-operation is included in aid (22).

Commitments refer to a firm obligation, expressed in writing and backed by the necessary funds, undertaken by an official donor to provide specified assistance to a recipient country or a multilateral organization (22). Recipients are defined by the CRS as all “developing countries” eligible to receive ODA. These include all “least developed countries” as defined by the United Nations and all LMICs defined by the World Bank, except any members of the G8, or members or agreed future members of the European Union (23).

Disbursements refer to the release of funds to or the purchase of goods or services for a recipient; by extension, the amount thus spent. Disbursements record the actual international transfer of financial resources, or of goods or services valued at the cost to the donor. In the case of activities carried out in donor countries, such as training, administration or public awareness programmes, disbursement is taken to have occurred when the funds have been transferred to the service provider or the recipient.

WHO and the United Nations Interagency Working Group set 17 population-based indicators to provide an overview of the global and national SRH situation (24). We divided these indicators into health facility level process/outcome indicators and health impact level indicators. Of the process/outcome indicators, we selected indicators that are routinely collected using country demographic health surveys conducted between 2002 and 2018. These include modern contraceptive prevalence rate (mCPR), unmet need for family planning (FP), antenatal care coverage (ANC) and percent of births attended by skilled health personnel. For impact we selected the mortality indicators, maternal mortality rate (MMR) and neonatal mortality rate (NMR), and added infant mortality rate (IMR) and under five mortality rate (U5MR).

### Data Collection

We downloaded ODA data on commitments and disbursements for all donors for population policies, programs and reproductive health for four sub-Sahara African countries; Kenya, Tanzania, Uganda and Zambia from the OECD CRS for a 16-year period (2002 to 2017) on 22nd September, 2019.

OECD-CRS database has eight parameters: donors, sectors, ODA flow, channels, amount type, flow type, type of aid, and unit of aid in US million dollars. We selected data for all 110 donors reporting onto the system to the four recipient countries. Under sectors we selected code 130 with data on population policies/ programs and reproductive health and took into consideration all its subgroups which included population policy and administrative management, family planning, sexually transmitted diseases control and personnel development. We used total ODA and we considered all the different channels of fund flows including the public sector, non-government organizations (NGOs) and civil society, public-private partnerships, multilateral organizations, teaching institutions, research institutions or think tanks. On amount type, we chose constant prices in US dollars (USD) which is the amount that is adjusted for the effects of inflation. Under flow types, we considered both commitments and disbursements. We selected all types of aid including budget support, core contribution and pooled programmes, project-type interventions and technical assistance. The selected data was then exported into Microsoft Excel spreadsheet.

We collected data on the SRH indicators from the Global Health Observatory (GHO) and DHS surveys accessed from DHS StatCompiler on 22nd September, 2019. The Global Health Observatory derives this data from the United Nations Inter Agency Group (UN IAG) for Child Mortality Estimates: Levels and Trends in Child Mortality, Report 2017 (Available from: http://www.childmortality.org). Data on the MMR was derived from the World Bank Database available at http://data.worldbank.org/indicators/sh.sta.mmrt. We selected the four countries (Kenya, Tanzania, Uganda and Zambia) and filtered available data which was for the period (2002-2017) that was then exported into Microsoft Excel.

### Data Analysis

We studied trends for donor commitments and disbursements of SRH aid for the period 2002–2017 to the four countries. We examined variations in: the commitments and disbursements over time by total value; the commitments and disbursements over time by different types of donors (we considered DAC countries, multilateral organizations, UN agencies and the World Bank which contributed 83.4% of funding to the four countries); and examined the commitments and disbursements over time to each of the four countries and by type of donors to each of the countries.

In a descriptive manner, we compared the time series data on donor aid disbursements to SRH indicators in each of the four countries.

## Results

### Total Donor Commitments for SRH Aid to the Countries

**Figure 1:**
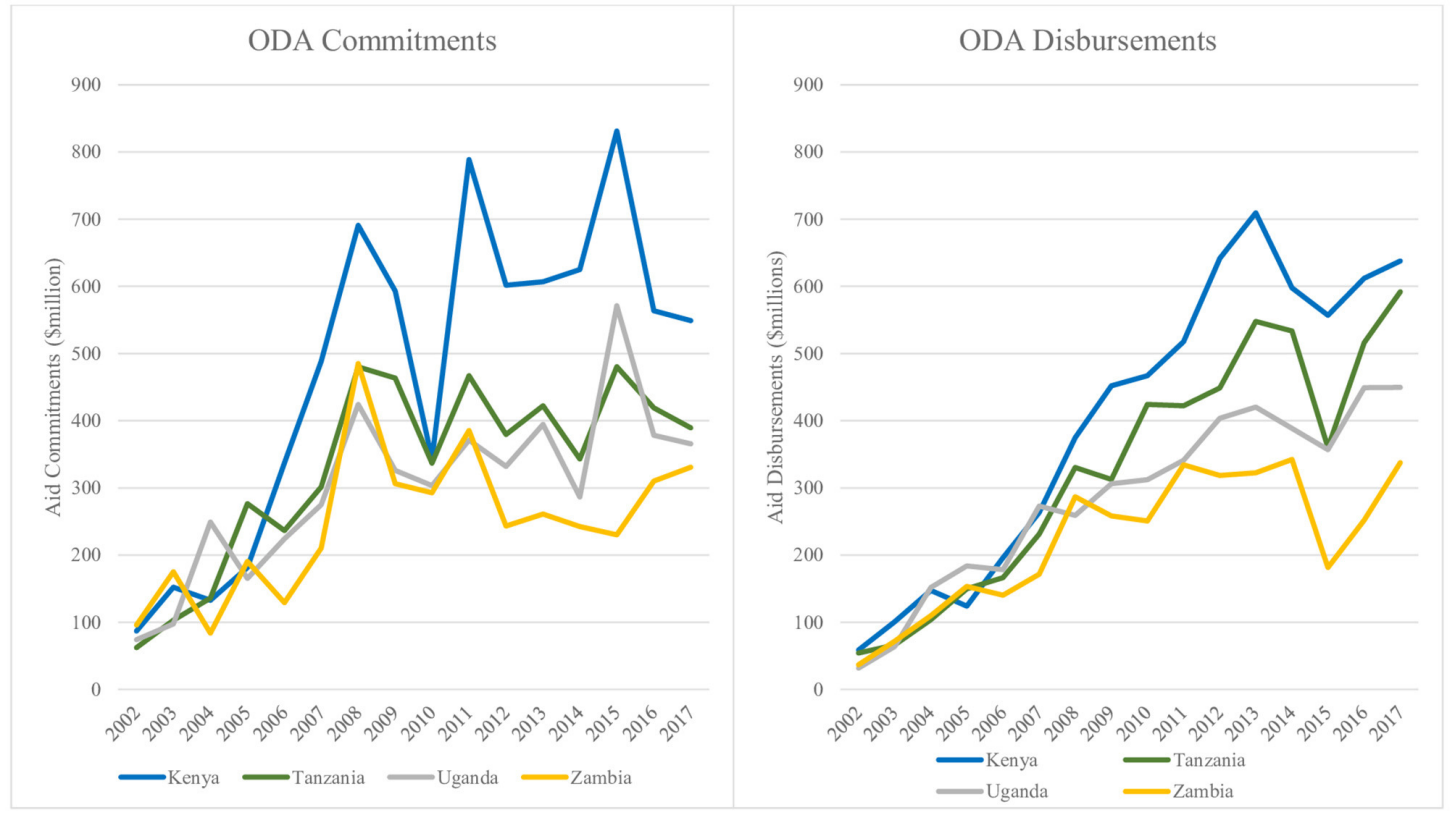
Trends in donor commitments and disbursements for SRH aid to countries 2002–2017 (in USD millions).

Total donor commitments for SRH to the four countries (Kenya, Tanzania, Uganda and Zambia) grew annually by 20% on average between 2002 and 2017 from USD 319.14 million to 1,635.05 million. There was an increase in commitments between 2002 and 2008 but thereafter there were fluctuations. The total amount of commitments equalled USD 21,678 million over the 16-year period. Kenya received the largest donor commitments totalling USD 7,571.24 million (35%) over the sixteen-year period, followed by Tanzania at 24% amounting to USD 5,296.66 million, Uganda at 22% amounting to USD 4,837.67 million and then Zambia being the lowest at 18% amounting to USD 3,972.04 million. Despite the general growth in commitments, there were year on year fluctuations over the period with a general decline in 2010. Figure 1 shows trends in donor commitments to the four countries.

DAC countries committed the largest proportion (82%) equivalent to USD 18,444.25 million over the sixteen-year period (2002-2017) followed by multilateral institutions, UN agencies and then the World Bank as shown in Annex 1.

**Annex 1:** SRH aid donor commitments and disbursements by donor type (2002–2017), amounts are expressed in million US dollars.

**Commitments**																	
	**2002**	**2003**	**2004**	**2005**	**2006**	**2007**	**2008**	**2009**	**2010**	**2011**	**2012**	**2013**	**2014**	**2015**	**2016**	**2017**	**TOTAL**
**DAC**																	
Kenya	78.553	99.658	125.05	175.536	252.222	426.107	640.742	586.829	298.035	675.744	595.509	600.713	402.017	622.324	514.439	546.697	6640.175
Tanzania	54.354	54.638	94.037	155.153	160.298	217.03	319.881	339.207	328.858	383.507	302.409	368.666	310.246	425.458	414.982	385.601	4314.325
Uganda	68.137	45.426	128.998	159.22	217.973	267.499	338.028	315.62	294.022	350.071	276.549	386.085	278.672	382.17	374.85	362.284	4245.604
Zambia	63.155	120.596	81.331	125.684	122.639	184.706	260.846	246.388	256.685	227.943	202.837	254.779	236.784	227.983	302.879	328.914	3244.149
**Total**	**264.199**	**320.318**	**429.416**	**615.593**	**753.132**	**1095.342**	**1559.497**	**1488.044**	**1177.6**	**1637.265**	**1377.304**	**1610.243**	**1227.719**	**1657.935**	**1607.15**	**1623.496**	
**Multilaterals**																	
Kenya	8.374	52.359	7.539	5.894	84.669	62.300	50.019	6.263886	45.101221	112.72033	6.186066	5.990998	223.14406	208.90446	49.119027	2.476	931.063
Tanzania	7.858	48.412	41.935	121.148	76.179	84.234	160.242	124.30244	7.868091	83.56549	76.996327	53.665792	32.820869	54.93998	4.191553	3.810	982.173
Uganda	5.898	52.208	120.021	6.155	6.078	7.408	86.305	10.239727	9.384738	21.034499	55.47317	8.428447	7.872573	188.95425	3.232976	3.368	592.066
Zambia	32.810	54.664	2.495	64.837	6.389	25.629	224.243	59.610526	36.159785	157.35712	40.471814	6.370723	5.474929	2.0751	7.403347	1.898	727.891
**Total**	**54.941**	**207.645**	**171.993**	**198.035**	**173.317**	**179.572**	**520.810**	**200.416**	**98.513**	**374.677**	**179.127**	**74.455**	**269.312**	**454.873**	**63.946**	**11.554**	
**UN**																	
Kenya	8.374	4.35	4.1	5.895	5.038	4.4	6.458	6.264	6.173	6.119	6.186	5.991	6.486	2.49	3.301	2.476	84.101
Tanzania	6.074	6.56	4.006	5.97	6.389	7.015	6.877	7.332	7.868	7.464	6.643	7.152	7.511	4.305	4.192	3.811	99.169
Uganda	5.899	8.175	5.079	6.155	6.078	7.408	9.285	10.24	9.385	8.005	9.247	8.309	7.873	3.653	3.233	3.369	111.393
Zambia	3.992	2.758	2.495	4.793	6.39	6.391	7.46	6.445	6.394	5.454	6.607	6.371	5.475	2.075	2.822	1.898	77.82
**Total**	**24.339**	**21.843**	**15.680**	**22.813**	**23.895**	**25.214**	**30.080**	**30.281**	**29.820**	**27.042**	**28.683**	**27.823**	**27.345**	**12.523**	**13.548**	**11.554**	
**World Bank**																	
Kenya	.	.	.	.	.	57.900	.	.	38.927	.	.	.	.	.	45.817	.	142.646
Tanzania	1.784	33.951	11.171	.	.	.	.	.	.	.	.	.	.	50.634	.	.	97.542
Uganda	.	.	33.515	.	.	.	.	.	.	.	.	.	.	.	.	.	33.515
Zambia	28.818	.	.	.	.	.	.	.	.	.	.	.	.	.	4.581	.	33.400
**Total**	**30.603**	**33.952**	**44.687**	**0.000**	**0.000**	**57.900**	**0.000**	**0.000**	**38.928**	**0.000**	**0.000**	**0.000**	**0.000**	**50.635**	**50.400**	**0.000**	
**Disbursements**																	
	**2002**	**2003**	**2004**	**2005**	**2006**	**2007**	**2008**	**2009**	**2010**	**2011**	**2012**	**2013**	**2014**	**2015**	**2016**	**2017**	**TOTAL**
**DAC**																	
Kenya	41.864	73.485	103.113	108.543	188.013	231.201	334.214	428.222	405.305	478.938	562.696	625.659	533.152	445.266	523.018	543.830	5626.526
Tanzania	44.696	51.298	90.207	86.502	111.964	160.574	213.178	243.162	325.650	340.390	342.201	371.138	396.040	323.0263	346.625	415.252	3861.909
Uganda	20.978	49.379	109.961	130.106	170.654	226.643	246.705	291.195	278.517	317.901	343.652	378.290	346.411	264.3558	336.094	400.685	3911.535
Zambia	30.824	61.773	67.213	104.188	109.668	131.055	196.729	206.357	207.678	248.153	243.417	246.251	273.766	176.9099	227.847	326.241	2858.076
**Total**	**138.364**	**235.935**	**370.495**	**429.341**	**580.301**	**749.474**	**990.827**	**1168.937**	**1217.152**	**1385.384**	**1491.968**	**1621.340**	**1549.370**	**1209.558**	**1433.585**	**1686.01**	
**Multilaterals**																	
Kenya	16.768	27.301	44.336	15.280	8.251	32.406	40.386	23.681	61.785	38.921	79.041	83.738	64.511	111.624	89.1062	93.855	830.998
Tanzania	9.438	14.947	13.552	63.094	54.215	70.908	117.075	69.308	98.581	81.662	106.502	176.519	137.384	37.167	169.560	176.624	1,396.544
Uganda	10.796	14.570	41.874	53.788	7.637	46.177	12.242	14.847	33.591	23.167	60.116	41.810	42.307	92.46	112.938	48.926	657.262
Zambia	5.904	9.988	42.4543	49.238	30.526	40.504	90.173	51.818	43.251	86.356	75.061	76.099	68.535	4.619	23.948	11.437	709.917
**Total**	**42.907**	**66.808**	**142.218**	**181.402**	**100.630**	**189.998**	**259.878**	**159.655**	**237.211**	**230.108**	**320.722**	**378.168**	**312.739**	**245.881**	**395.554**	**330.843**	
**UN**																	
Kenya	8.374	4.350	4.100	5.894	5.038	4.399	6.457	6.691	6.578	6.599	7.143	6.285	6.486	7.470	7.781	5.145	98.798
Tanzania	6.074204	6.559978	4.006439	5.969597	6.388663	7.014642	6.876169	7.550227	7.926127	7.464311	6.642515	7.152426	7.510546	7.328398	6.930842	4.784	106.180
Uganda	5.898	8.175	5.079	6.155	6.078	7.408	9.285	10.239	9.384	8.004	9.246	8.309	7.872	8.276	6.896	6.126	122.437
Zambia	3.99	2.758	2.495	4.730	6.389	6.389	7.459	7.623	6.394	5.453	6.607	6.370	5.474	4.287	4.827	3.087	84.343
**Total**	**24.339**	**21.844**	**15.681**	**22.750**	**23.895**	**25.212**	**30.079**	**32.105**	**30.283**	**27.522**	**29.640**	**28.118**	**27.345**	**27.364**	**26.436**	**19.143**	
**World Bank**																	
Kenya	8.271	12.059	12.676	7.257	0.992	3.215	13.729	0.359	26.427	24.885	15.327	10.664	1.862	4.233	0	4.088	146.051
Tanzania	3.066	4.552	0.915	6.160	5.775	16.061	9.113	6.756	0.896	0	0	0	0	0	12.843	11.168	77.311
Uganda	3.455	4.163	5.796	37.420	1.332	-0.049	0	0	0	0	0	0	0	0	0	0	52.120
Zambia	1.771	3.741	10.341	9.726	5.761	15.227	1.721	0.0173	0	0	0	0	0	0	0	0.647	48.957
**Total**	**16.566**	**24.517**	**29.730**	**60.566**	**13.862**	**34.455**	**24.565**	**7.134**	**27.324**	**24.885**	**15.327**	**10.665**	**1.863**	**4.233**	**12.844**	**15.904**	

DAC countries committed the highest amount (36% of their commitments) to a tune of USD 5,989.29 million to Kenya. Tanzania took the largest commitment of funds (USD 960.69 million, 30%) from multilateral donors. Uganda received the largest commitment of funds (USD 106.10 million, 30%) from UN agencies whereas the World Bank also committed most of its funds (USD 139.95 million, 46%) to Kenya. In contrast, Tanzania, Uganda and Zambia did not receive any commitments for SRH funds from the World Bank between 2005 and 2014. See table in Annex 1 for details.

### Total Donor Aid Disbursements to Countries

The total disbursements to the four countries over the 16-year period were USD 19,852.92 million. The overall disbursement rate over the sixteen-year period was 93%. Disbursements grew over time rising from USD 181.27 million in 2002 to 1,999.51 million in 2013, but thereafter reduced to 1,455.43 million in 2015 and rising to 2,016.85 million in 2017 at an average annual growth rate of 21%. In contrast with the commitments, there was a steady increase in disbursements until 2013 and 2014 for Zambia and a drop in 2015 from where disbursements then started to rise slowly. Kenya received the largest donor disbursements totalling USD 6,457.52 million (33%) over the sixteen-year period, followed by Tanzania at 26% amounting to USD 5,258.61 million, Uganda at 23% amounting to USD 4,568.79 million and then Zambia being the lowest at 18% amounting to USD 3,567.99 million. Despite the general growth in disbursements, there was a general decline between 2013 and 2015 before picking up in 2016. Trends in donor aid disbursements to the four countries are shown in Figure 1.

The highest donor disbursement over the sixteen-year period (2002–2017) was from DAC countries comprising 79% of the total and rising from USD 138.36 million in 2002 to USD 1,686.01 million in 2017. Multilateral funders followed the DAC countries contributing 17% of disbursements. United Nation agencies and the World Bank contributed 2% each. Trends in donor aid disbursements for SRH by donor type in the four countries are shown in Annex 1.

Kenya was the biggest recipient from DAC countries getting 35% of funds worth USD 5,626.52 million over the period 2002–2017. Tanzania took the largest proportion (39%) of funds (USD 1,396.54 million) from multilateral donors; Uganda received the largest proportion 30% of funds worth USD 122.43 million from UN agencies whereas the World Bank also provided most (45%) of its funds (USD 146.05 million) to Kenya. This is detailed in table in Annex 1.

### Country Specific Donor aid Commitments and Disbursements

**Figure 2:**
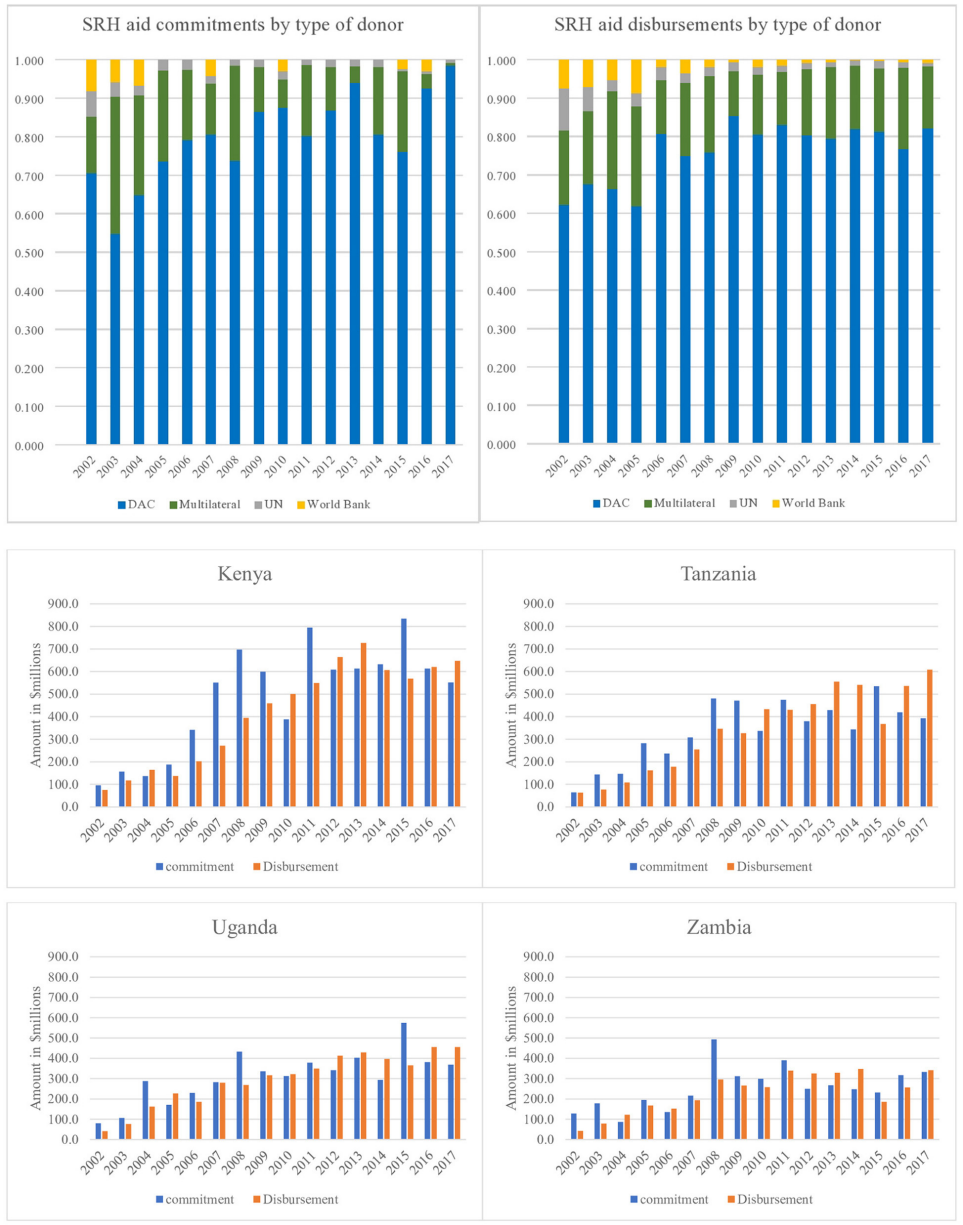
Country specific comparisons in donor aid commitments and disbursements, 2002–2017 (USD millions).

Figure 2 highlights the trends i1n the donor aid commitments and disbursements to each of the four countries. The trends show that the commitments and disbursements grew mostly in line overtime but peaks in commitments were not reflected in the disbursements. While Kenya received most aid, it also had most fluctuations between amounts committed and disbursed. For Kenya 86% of commitments were disbursed compared to 100% of commitments for Tanzania, 95% for Uganda and 91% for Zambia over the total study period.

### Comparison of Trends in Donor Disbursements to SRH Indicators

**Figure 3:**
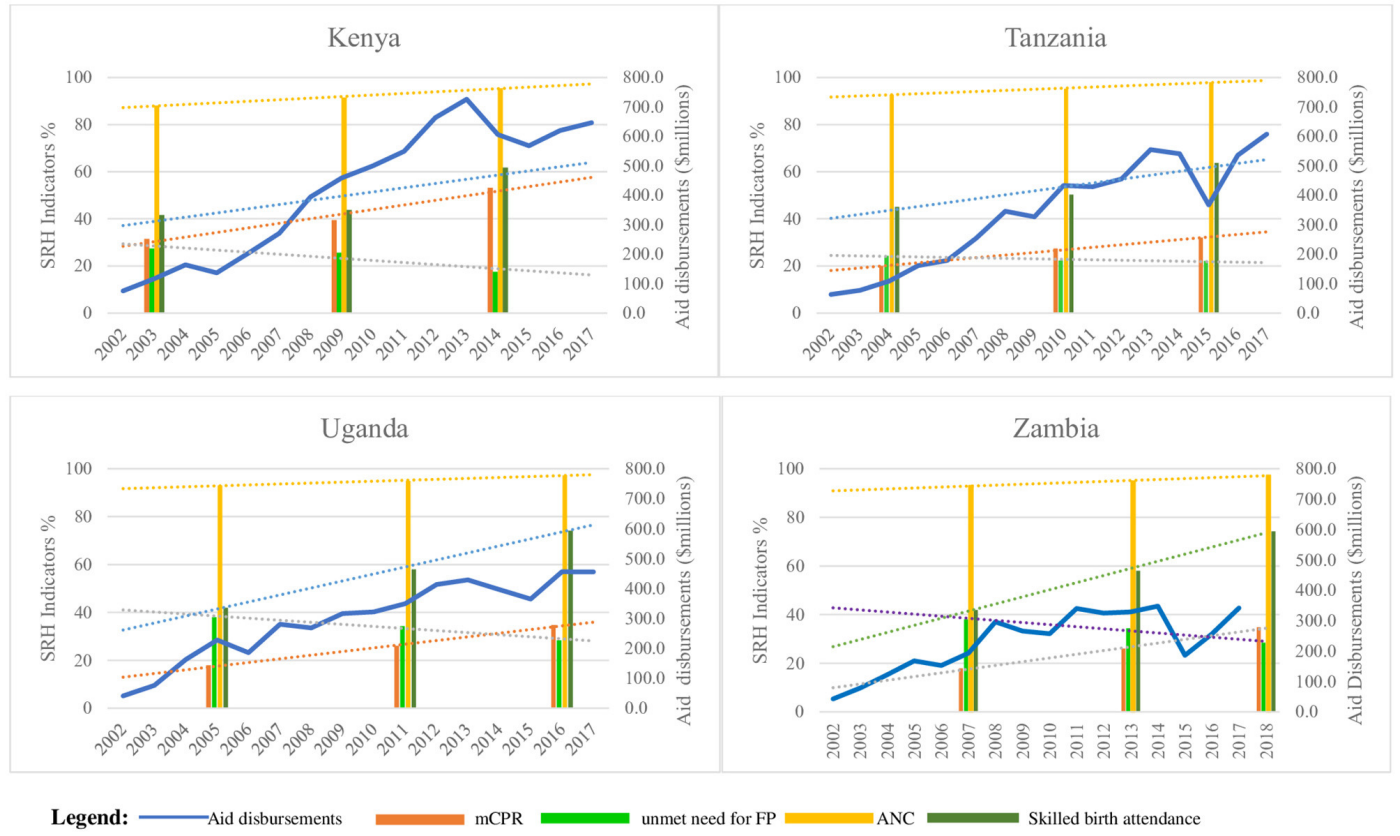
Comparison of disbursements with selected SRH health facility level process/outcome indicators.

**Figure 4:**
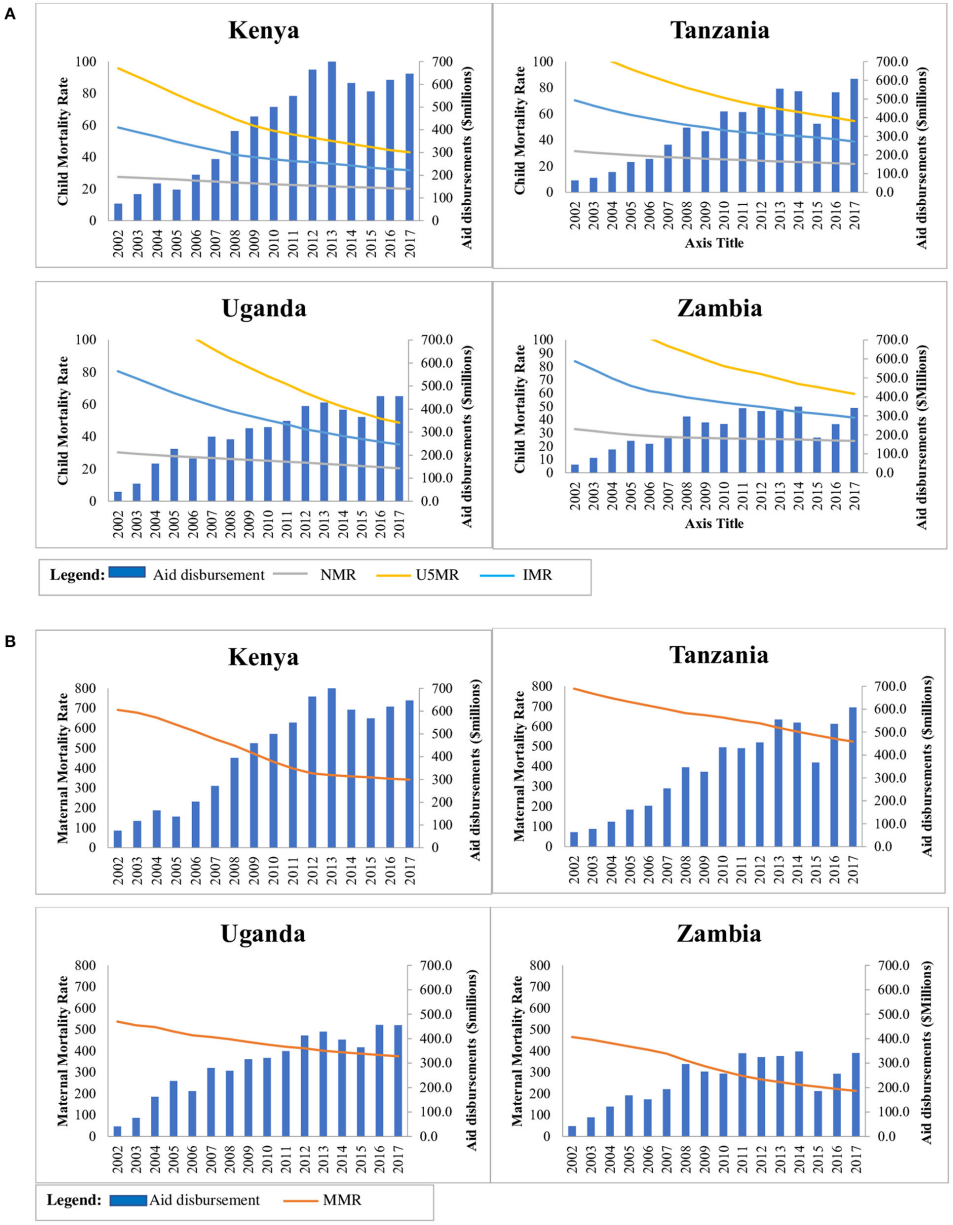
**(A)** Comparison of aid disbursements with neonatal, under five and child mortality rates. **(B)** Comparison of aid disbursements with maternal mortality rates.

Figure 3 shows SRH health facility level process/outcome indicators which showed improvement over the 16-year period across the four countries. ANC improved and remained very high, mCPR increased with most pronounced increase observed in Kenya, and unmet need for FP reduced mostly in Kenya. Tanzania was much slower in improvement in the indicators. Skilled birth attendance increase was most pronounced in Uganda and Zambia. Improvement in SRH impact indicators (Figure 4) were most pronounced for Kenya. U5MR and IMR dropped markedly across the four countries but reduction in NMR was slow. MMR dropped across the four countries with Kenya having the most pronounced improvement. The rise in donor aid disbursements between 2005 and 2017 aligned with improved SRH outcome and impact indicators but not enough to meet SDG targets. Impact indicators reduced majorly between 2002 and 2005 and slowed thereafter especially for under five mortality.

## Discussion

Between 2002 and 2017, donor commitments for SRH aid to the four sub-Saharan countries of Kenya, Tanzania, Uganda and Zambia grew annually by 20% on average while disbursements grew at an average annual growth rate of 21%. DAC countries committed and disbursed the largest proportion (82 and 79%, respectively) over the sixteen-year period. Kenya received the largest proportion of aid (33%) and was most favored by DAC donors. Whereas, overall 93% of committed aid to SRH was disbursed over the 16 year period, there were year on year fluctuations in both commitments and disbursement. The study showed improvements in both SRH process/outcome indicators and impact indicators.

The trend of growth in donor aid observed in this study is in line with studies done at a global scale (18, 21, 25–29). There was an increase in both commitments and disbursements between 2002 and 2008. During 2009 to 2013 disbursements continued to grow although commitments declined. Toward the end of the Millennium Development Goals era between 2013 and 2015, a decline was observed in both commitments and disbursements for donor aid to the four countries. However, there was an increase in 2016 at the start of the SDGs era. These changes may point to some unpredictability of aid.

The peaks and dips in aid that are observed are not markedly erratic which may reinforce John Hudson's assertion that aid to health is one of the least volatile (30). However, Kenya had the largest fluctuation between funds committed and those disbursed. When donors do not disburse what they commit, it affects the recipient governments' ability to plan and therefore impacts on results as was noted by Arregoces et al. (21). Recipient countries should therefore cautiously rely on aid and track volatility in aid provided.

While there was improvement in both SRH outcome and impact indicators alongside growth in donor aid over the 16-year period, the SRH impact indicators are not reducing fast enough to meet SDG targets (2, 4). Kenya, which received most funds, also had the most promising SRH indicators. Zambia on the other hand received the least SRH aid over the period and with its population that is less than half of any of the other three countries, is struggling with SRH indicators of a similar magnitude (4–8). LMICs will require more concerted efforts to avert future maternal and child mortality.

The disbursements by the different donors showed countries of preference. DAC countries and the World Bank provided most aid to Kenya; Tanzania received most funds from multilateral donors whereas Uganda was most preferred by UN agencies. Donors have preferred countries to provide aid based on strategic interests. The magnitude of aid may differ based on various reasons, for example; delays in project implementation, emergencies that call for immediate support interventions, sometimes donor countries have realized more or less than expected growth and therefore have more or less aid available, other times there are changes in donor political environment.

The preference for some countries by donors may also not be targeted to recipient national priorities or countries with most need as noted by Grollman et al. and other studies (26, 31). However, what is clear is the need for more funding to meet SDG3 targets (10) and therefore more deliberate targeting of funding to country needs and priority interventions is required (32–34). Countries have an obligation to the United Nations to spend a target of 0.7% of their gross national income (GNI) on international aid (35). Bilateral aid is a reflection of strategic interests of donors and is driven by variables that include: an obligation to protect human rights, dignity and solidarity; trade and economic relations with recipient countries; political interests including creating stability in poor countries to reduce migration; level of transparency and accountability within recipient governments (36, 37). However, politics is the ultimate determinant (38–40). As countries develop, donor countries prefer to transition from aid to trade. LMICs therefore ought to progressively move away from reliance on donor support and increase country ownership of health needs by consistently improving domestic investments in SRH (41, 42), as reflected in the 2017 Tokyo declaration on Universal Health Coverage (43).

To meet the aspirations of the United Nations (UN) Secretary-General's *Global Strategy for Women's, Children's and Adolescents' Health, 2016–2030* (9) and the Tokyo Declaration to Universal Health Coverage (43), more efforts will be required to mobilize governments, donors and other stakeholders to ensure sustained funding to SRH (42). This is especially important in the light of slower improvement in some of the SRH indicators in the latest years. Aid has been shown in other studies to have positive long-term effect on health and on development (44–46). Also, Dieleman et al. note that in the near term, increased domestic spending on health alone is unlikely to cover the gaps to meet the ambitious health goals laid out in the SDGs (47). Therefore, increased funding to DAH is required and should be in accordance with principles of the Paris Declaration on Aid Effectiveness.

SRHR investments are critical to people's well-being, the prosperity and resilience of families, communities and nations (17). These investments are cost-effective and cost-saving, freeing resources for investment in other development priorities with high pay-offs for equality and equity. Regardless of the demonstrated returns on investments, underfunding of SRHR still persists. This is a contributing factor to why the core goal of achieving universal access to sexual and reproductive services adopted by 179 governments at the International Conference on Population and Development (ICPD 1994) remains unfulfilled (17).

The Organization for Economic Co-operation and Development's Development Assistance Committee (OECD DAC) is one of the most comprehensive tracking platforms for resource flows. The OECD CRS information has been recorded, in one form or another, since 1967. It is relatively complete in terms of bilateral aid commitments since 1995. Even taking into account changes in definitions, the time series information is the most stringent and validated database on aid flows that currently exists (48). In addition, the database provides for accuracy of data as it ensures that accurate and comparable measurements of donor outflows can be derived (48).

As shown by other similar studies, donor reporting to the CRS has improved over time (49). The CRS is limited by the accuracy, completeness, consistency, and timeliness of donor reports to improve data in the system which also affect study is limited. It is important that more efforts are made to ensure improvement of data under the CRS. It is also important to note that the study only describes donor funding for SRH and how it compares with some of the most critical SRH indicators. Statistical assessment of the relation between disbursements and SRH indicators was not carried out since there are many factors which influence these indicators. The study does not presuppose that aid can be independent of both government (domestic) funding and out-of-pocket payments (49). As is noted in other studies, this study also does not explain variation or timeliness in donor aid to different countries and therefore further research is needed (50). However, the strength of this study is that it zooms in on some of the specific countries with most need, begins to assess predictability of aid by assessing commitments and disbursements to add to the body of knowledge on accountability of donor aid for SRH. Reporting on the time when both commitments and disbursements are made in CRS will help strengthen arguments around predictability of aid which is important for recipient country planning (41).

## Conclusion

Donor commitments for SRH aid grew on average by 20% annually while disbursements grew by 21% annually between 2002 and 2017. There was improvement in SRH indicators alongside growth in donor aid but improvement is slow to meet SGD targets. There were year on year fluctuations in both commitments and disbursements. Unpredictability and insufficiency of donor aid may be disruptive to country planning and may lead to missing of global targets on SRH. Donors and LMICs should increase domestic investments in order to meet global SRHR targets.

## Data Availability Statement

The original contributions presented in the study are included in the article/supplementary material, further inquiries can be directed to the corresponding author/s.

## Author Contributions

DK conceptualized the project and undertook data analysis and wrote the first draft of the manuscript. CA, MM, HH, TR, HL, and AM-T revised the manuscript and critically reviewed its contents. CA contributed to data collection and data analysis. AM-T and HH critically reviewed the manuscript, provided comments and guidance on all drafts of manuscript. All authors contributed to the article and approved the submitted version.

## Conflict of Interest

The authors declare that the research was conducted in the absence of any commercial or financial relationships that could be construed as a potential conflict of interest.

## Abbreviations

SRHR: sexual and reproductive health and rights
DAC: development assistance cooperation
LMICs: low- and middle-income countries
SDGs: sustainable development goals
DAH: development assistance for health
ODA: official development assistance
OECD CRS: organization for economic co-operation and development's assistance creditor reporting system.
