# Supplementary material for: Donor Commitments and Disbursements for Sexual and Reproductive Health Aid in Kenya, Tanzania, Uganda and Zambia
Source: Front Public Health. 2021 Apr 20;9:645499. doi: 10.3389/fpubh.2021.645499 (PMC8093629; doi:10.3389/fpubh.2021.645499)
Supplement: Supplementary file 1 [file Data_Sheet_1.pdf]

Annex 2

SRH aid donor commitments and disbursements by donor type (2002-2017), amounts are expressed in million US dollars

**Commitments**																	
	**2002**	**2003**	**2004**	**2005**	**2006**	**2007**	**2008**	**2009**	**2010**	**2011**	**2012**	**2013**	**2014**	**2015**	**2016**	**2017**	**TOTAL**
**DAC**																	
Kenya	78.553	99.658	125.05	175.536	252.222	426.107	640.742	586.829	298.035	675.744	595.509	600.713	402.017	622.324	514.439	546.697	6640.175
Tanzania	54.354	54.638	94.037	155.153	160.298	217.03	319.881	339.207	328.858	383.507	302.409	368.666	310.246	425.458	414.982	385.601	4314.325
Uganda	68.137	45.426	128.998	159.22	217.973	267.499	338.028	315.62	294.022	350.071	276.549	386.085	278.672	382.17	374.85	362.284	4245.604
Zambia	63.155	120.596	81.331	125.684	122.639	184.706	260.846	246.388	256.685	227.943	202.837	254.779	236.784	227.983	302.879	328.914	3244.149
**Total**	**264.199**	**320.318**	**429.416**	**615.593**	**753.132**	**1095.342**	**1559.497**	**1488.044**	**1177.6**	**1637.265**	**1377.304**	**1610.243**	**1227.719**	**1657.935**	**1607.15**	**1623.496**	
**Multilaterals**																	
Kenya	8.374	52.359	7.539	5.894	84.669	62.300	50.019	6.263886	45.101221	112.72033	6.186066	5.990998	223.14406	208.90446	49.119027	2.476	931.063
Tanzania	7.858	48.412	41.935	121.148	76.179	84.234	160.242	124.30244	7.868091	83.56549	76.996327	53.665792	32.820869	54.93998	4.191553	3.810	982.173
Uganda	5.898	52.208	120.021	6.155	6.078	7.408	86.305	10.239727	9.384738	21.034499	55.47317	8.428447	7.872573	188.95425	3.232976	3.368	592.066
Zambia	32.810	54.664	2.495	64.837	6.389	25.629	224.243	59.610526	36.159785	157.35712	40.471814	6.370723	5.474929	2.0751	7.403347	1.898	727.891
**Total**	**54.941**	**207.645**	**171.993**	**198.035**	**173.317**	**179.572**	**520.810**	**200.416**	**98.513**	**374.677**	**179.127**	**74.455**	**269.312**	**454.873**	**63.946**	**11.554**	
**UN**																	
Kenya	8.374	4.35	4.1	5.895	5.038	4.4	6.458	6.264	6.173	6.119	6.186	5.991	6.486	2.49	3.301	2.476	84.101
Tanzania	6.074	6.56	4.006	5.97	6.389	7.015	6.877	7.332	7.868	7.464	6.643	7.152	7.511	4.305	4.192	3.811	99.169
Uganda	5.899	8.175	5.079	6.155	6.078	7.408	9.285	10.24	9.385	8.005	9.247	8.309	7.873	3.653	3.233	3.369	111.393
Zambia	3.992	2.758	2.495	4.793	6.39	6.391	7.46	6.445	6.394	5.454	6.607	6.371	5.475	2.075	2.822	1.898	77.82

**Total**	**24.339**	**21.843**	**15.680**	**22.813**	**23.895**	**25.214**	**30.080**	**30.281**	**29.820**	**27.042**	**28.683**	**27.823**	**27.345**	**12.523**	**13.548**	**11.554**	
**World Bank**																	
Kenya	..	..	..	..	..	57.900	..	..	38.927	..	..	..	..	..	45.817	..	142.646
Tanzania	1.784	33.951	11.171	..	..	..	..	..	..	..	..	..	..	50.634	..	..	97.542
Uganda	..	..	33.515	..	..	..	..	..	..	..	..	..	..	..	..	..	33.515
Zambia	28.818	..	..	..	..	..	..	..	..	..	..	..	..	..	4.581	..	33.400
**Total**	**30.603**	**33.952**	**44.687**	**0.000**	**0.000**	**57.900**	**0.000**	**0.000**	**38.928**	**0.000**	**0.000**	**0.000**	**0.000**	**50.635**	**50.400**	**0.000**	
**Disbursements**																	
	**2002**	**2003**	**2004**	**2005**	**2006**	**2007**	**2008**	**2009**	**2010**	**2011**	**2012**	**2013**	**2014**	**2015**	**2016**	**2017**	**TOTAL**
**DAC**																	
Kenya	41.864	73.485	103.113	108.543	188.013	231.201	334.214	428.222	405.305	478.938	562.696	625.659	533.152	445.266	523.018	543.830	5626.526
Tanzania	44.696	51.298	90.207	86.502	111.964	160.574	213.178	243.162	325.650	340.390	342.201	371.138	396.040	323.0263	346.625	415.252	3861.909
Uganda	20.978	49.379	109.961	130.106	170.654	226.643	246.705	291.195	278.517	317.901	343.652	378.290	346.411	264.3558	336.094	400.685	3911.535
Zambia	30.824	61.773	67.213	104.188	109.668	131.055	196.729	206.357	207.678	248.153	243.417	246.251	273.766	176.9099	227.847	326.241	2858.076
**Total**	**138.364**	**235.935**	**370.495**	**429.341**	**580.301**	**749.474**	**990.827**	**1168.937**	**1217.152**	**1385.384**	**1491.968**	**1621.340**	**1549.370**	**1209.558**	**1433.585**	**1686.01**	
**Multilaterals**																	
Kenya	16.768	27.301	44.336	15.280	8.251	32.406	40.386	23.681	61.785	38.921	79.041	83.738	64.511	111.624	89.1062	93.855	830.998
Tanzania	9.438	14.947	13.552	63.094	54.215	70.908	117.075	69.308	98.581	81.662	106.502	176.519	137.384	37.167	169.560	176.624	1,396.544
Uganda	10.796	14.570	41.874	53.788	7.637	46.177	12.242	14.847	33.591	23.167	60.116	41.810	42.307	92.46	112.938	48.926	657.262
Zambia	5.904	9.988	42.4543	49.238	30.526	40.504	90.173	51.818	43.251	86.356	75.061	76.099	68.535	4.619	23.948	11.437	709.917
**Total**	**42.907**	**66.808**	**142.218**	**181.402**	**100.630**	**189.998**	**259.878**	**159.655**	**237.211**	**230.108**	**320.722**	**378.168**	**312.739**	**245.881**	**395.554**	**330.843**	

**UN**																
Kenya	8.374	4.350	4.100	5.894	5.038	4.399	6.457	6.691	6.578	6.599	7.143	6.285	6.486	7.470	7.781	5.145	98.798
Tanzania	6.074204	6.559978	4.006439	5.969597	6.388663	7.014642	6.876169	7.550227	7.926127	7.464311	6.642515	7.152426	7.510546	7.328398	6.930842	4.784	106.180
Uganda	5.898	8.175	5.079	6.155	6.078	7.408	9.285	10.239	9.384	8.004	9.246	8.309	7.872	8.276	6.896	6.126	122.437
Zambia	3.99	2.758	2.495	4.730	6.389	6.389	7.459	7.623	6.394	5.453	6.607	6.370	5.474	4.287	4.827	3.087	84.343
**Total**	**24.339**	**21.844**	**15.681**	**22.750**	**23.895**	**25.212**	**30.079**	**32.105**	**30.283**	**27.522**	**29.640**	**28.118**	**27.345**	**27.364**	**26.436**	**19.143**	
**World Bank**																
Kenya	8.271	12.059	12.676	7.257	0.992	3.215	13.729	0.359	26.427	24.885	15.327	10.664	1.862	4.233	0	4.088	146.051
Tanzania	3.066	4.552	0.915	6.160	5.775	16.061	9.113	6.756	0.896	0	0	0	0	0	12.843	11.168	77.311
Uganda	3.455	4.163	5.796	37.420	1.332	-0.049	0	0	0	0	0	0	0	0	0	0	52.120
Zambia	1.771	3.741	10.341	9.726	5.761	15.227	1.721	0.0173	0	0	0	0	0	0	0	0.647	48.957
**Total**	**16.566**	**24.517**	**29.730**	**60.566**	**13.862**	**34.455**	**24.565**	**7.134**	**27.324**	**24.885**	**15.327**	**10.665**	**1.863**	**4.233**	**12.844**	**15.904**	
